# PCR and Microscopic Identification of Isolated *Leishmania tropica* from Clinical Samples of Cutaneous Leishmaniasis in Human Population of Kohat Region in Khyber Pakhtunkhwa

**DOI:** 10.1155/2014/861831

**Published:** 2014-04-01

**Authors:** Nasser M. Abd El-Salam, Sultan Ayaz, Riaz Ullah

**Affiliations:** ^1^Arriyadh Community College, King Saud University, P.O. Box 28095, Riyadh 11437, Saudi Arabia; ^2^Department of Zoology, Kohat University of Science and Technology, Kohat 26000, Pakistan; ^3^Department of Chemistry, Government College Ara Khel, FR Kohat, Khyber Pakhtunkhwa 26000, Pakistan

## Abstract

*Leishmania tropica* was isolated from the clinical patients of cutaneous leishmaniasis in rural community of Kohat district in Khyber Pakhtunkhwa province and was identified through PCR, microscopy, and culture techniques. A total of 113 samples from the clinical patients were examined through PCR, microscopy, and culture which showed 87.61% (99/113), 53.98% (61/113), and 46.90% (53/113) prevalence. During the study, 186 bp *Leishmania tropica* was identified through PCR. Thus the sensitivity of PCR is very high as compared to the conventional techniques.

## 1. Introduction

Leishmaniasis is a disease caused by* Leishmania* parasite and transmitted to mammals and human beings by Phlebotomine sand flies and it causes skin infections [1, 2]. Twenty-one species of* Leishmania* have been reported to cause human infection [1]. Each year, 1.5–2 million new cases are reported and 70,000 deaths occurred. The number of disease and death cases showed about 2.4 million people affected throughout the world [3].* Leishmaniasis* can produce various symptoms in mammalian host depending on the host genetic makeup and species of the* Leishmania* parasite [4].

Approximately, 90% of the cases of the cutaneous leishmaniasis were observed in Iran, Afghanistan, Pakistan, Saudi Arabia, Brazil, Peru, and Syria [5]. Cutaneous leishmaniasis is a common infection that is endemic in many regions of Pakistan [6–8]. It is not a cause of mortality but can cause morbidity and social isolation due to its disfiguring complications. The lesions are mostly found on the exposed areas of the skin [6, 9, 10]. The lesion or ulcer leaves a scar on infected area [11]. Secondary bacterial or fungal infection of the ulcers causes increased tissue destruction and disfiguring of the skin [9].

Several techniques have been described for the identification of* Leishmania* at the molecular level. These techniques include sequence analysis of multicopy genes, restriction fragment length polymorphism, inter genic spacer regions, DNA fingerprinting, polymerase chain reaction (PCR), and randomly amplified polymorphic DNA [12–15].

The accurate identification and diagnosis which are concerned with epidemiology, clinical finding, and management and treatment of the patient must be based on molecular diagnosis. Among these multilocus enzyme electrophoresis and cytochrome B gene sequencing is the gold slandered for diagnosis and identification; however, DNA based techniques are being used frequently. The PCR and sequencing of cytochrome B gene were established for species identification [16]. Many studies have been conducted in Pakistan where the main causative agents in southern dry area were* Leishmania major* and* Leishmania tropica* [17].

A comprehensive need assessment is required to devise public health strategies for an effective prevention of this rapidly spreading infection, particularly in the Khyber Pakhtunkhwa. Observing the gravity of the situation where many cases of skin ulcers and nonhealing lesion were diagnosed as cutaneous leishmaniasis in the local Pakistani community [18]. Keeping in view the importance and health hazard of the disease, the present research was designed to focus on the isolation and identification of the local strain of cutaneous leishmaniasis infecting the population of the present community and to compare conventional and PCR methods in detection of cutaneous leishmaniasis.

## 2. Materials and Methods

### 2.1. Area of Intervention

**Figure 1:**
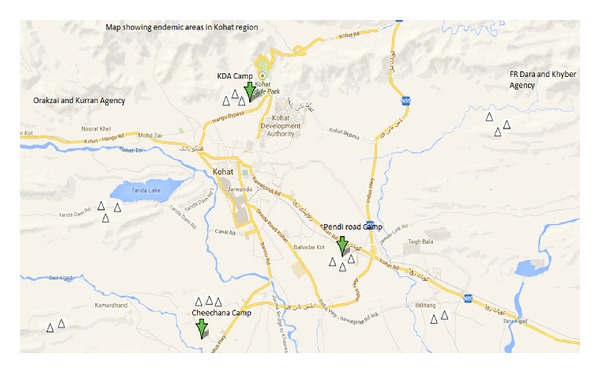


Samples were collected from the lesions of a patient with clinical suspected cutaneous leishmaniasis in rural communities in Kohat region of Khyber Pakhtunkhwa province (Figure 1). It is located at 33°35′13 N, 71°26′29 E with an altitude of about 489 meters above sea level and the total area is 2973 kilometers having population of 562640 individuals with an annual growth rate of 3.26%. The climate of the area is usually dry, extremely hot in summer and extremely cold and dry in winter. The average rainfall is about 400 mm. The Kohat region hosted more than 5 million IDPs in 2010, who migrated from military operated areas of tribal belt and Afghan refugees camp operating from the year 1981, which were the main source of CL migration from Afghanistan to the areas. The study was carried out from October 2010 to June 2011.

### 2.2. Sample Collection

Specimens were collected from* Leishmania* infected patients of cutaneous leishmaniasis. These lesions were cleaned with 70% alcohol. The skin scrapings were made with the help of scalpel in one direction till oozing out of the blood from the lesion and an incision was given mostly in the inflamed border of lesion. A smear of dermal tissue scrapings was collected from each patient. The smear samples were prepared and stained with Giemsa and then were seen under the microscope (100x) for presence of amastigote and the rest of the scraping samples were mixed with buffer at pH 7.2 and kept in sterile Eppendorf tube for future processes.

### 2.3. Cultivation and Isolation of Parasite

The culture medium RPMI-64 (Gibco, USA) was prepared for the purpose of the culturing of the* Leishmania* parasite from the samples. We dissolved the 0.3 g/30 mL of medium RPMI-64 in distilled water (10.43 g/1000 mL of distilled water) and distributed it amongst the 10 vials of bijou bottle, each having 3 mL of the dissolved media with supplemented 10% fetal bovine serum. The antibiotic consisting of penicillin G and kanamycin were mixed in cultural media to avoid bacterial contamination. The bijou bottles were placed in ice jar and were taken to the field where we collect the samples from the identified suspected person. The skin scrapings were directly mixed from the lesion to each bottle in ice jar and transported to the Department Lab of Kohat University of Science and Technology, Kohat. Then the medium was incubated at 26°C in incubator (Memmert Type INB500, Germany) and was examined at the different life stages of* Leishmania tropica* and was identified under 100x magnification of microscope for every 24 hours till up to 10 days.

### 2.4. DNA Extraction

The samples were subjected to DNA extraction by using GF-1 kit (Vivantis) as per the manufacturer protocol and extracted DNA were stored at −86°C for further process.

### 2.5. DNA Amplification

For detection of leishmania parasites, PCR was performed. A reaction mixture was prepared containing 5 *μ*L buffer (10X PCR buffer), dNTPs 5 *μ*L (500 *μ*M), MgCl_2_ (25 *μ*m), 5 *μ*L Primers (Forward 5-TTTCTTGGATGGGTTTCTGG-3 and Reverse 3-CAACACCAACGTAAGCGTAAC-3 of kDNA region), target DNA 5 *μ*L, 0.3 uni of taq. DNA polymerase and add deionized water up to 50 *μ*L. After an initial denaturation at 92°C for 3 minutes. PCR was performed with 35 cycles of denaturation (92°C, 1 minute), annealing 50°C and polymerization at (72°C, 1 minute). In the last stage extension at 72°C for 7 min and holding at 4°C for unlimited time, the designed program was saved as CL PCR.

### 2.6. Gel Electrophoreses

10 *μ*L of PCR product mixture was mixed with 5 *μ*L loading dye and loaded in a gel and 0.5 *μ*L ethidium bromide was added and poured into gel tray and combs were fixed. Combs were removed after gel was formed. Gel tray was placed in gel tank containing 1000 mL 0.5X TBE buffer. 15 *μ*L of each sample was loaded in the wells and 15 *μ*L of DNA ladder (100 bp) in separate well. The gel was run for 25 min at voltage of 120 volts and 500 ampere current. Gel was then examined by UV transilluminator.

### 2.7. Sensitivity and Specificity of Diagnostic Techniques

The sensitivity and specificity of PCR, microscopy, and culture technique were calculated by the following methods:

(1) Sensitivity =No.  of  True  PositiveNo.  of  True  Positive+No.  of  False  Negative,Specificity =No.  of  True  NegativeNo.  of  True  Negative+No.  of  False  Positive.

## 3. Results and Discussion

The productivity and the potential isolation of* Leishmania* amastigotes in RPMI-1640 media were assessed during the present study for evaluating the efficiency in the diagnosis and identification of the promastigotes and other life stages of the parasite.* Leishmania* samples were obtained from ten different suspected lesions of the patients. It was observed that the parasites were produced in seven bijou cultural bottles of RPMI-1640 media except in the three culture bottles. The results of cultivation, microscopic examination, and PCR of the materials were obtained from the patients having suspected CL lesions (Tables 1 and 2).

**Figure 2:**
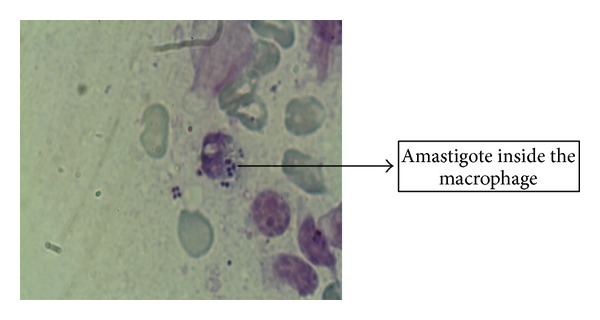
Giemsa stain image showing the amastigote form of* Leishmania* inside macrophages.

**Figure 3:**
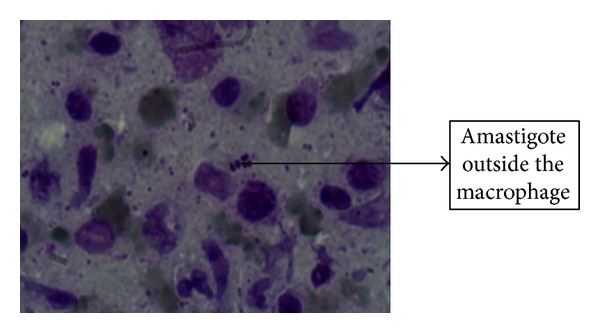
Giemsa stain image showing the amastigote form of* Leishmania* outside macrophages.

**Figure 4:**
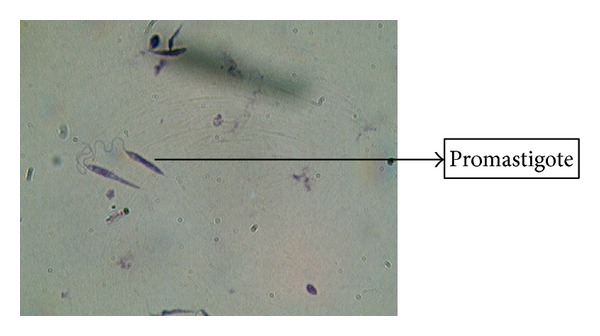
Giemsa stain image showing the promastigotes form of* Leishmania* in RPMI culture media.

**Figure 5:**
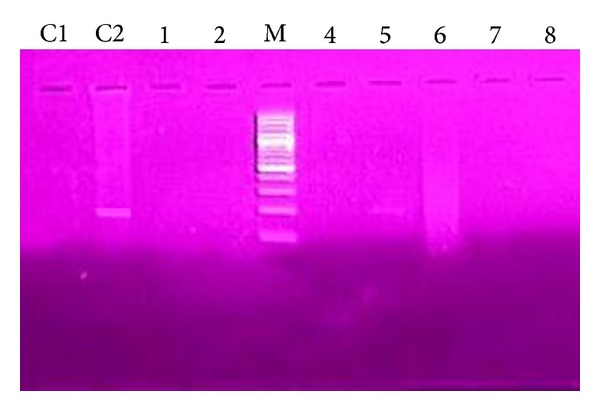
M: marker 100 bp, C1: negative control, C2: positive control, and 5: positive sample showing 186 bp.

No amastigote was observed in the other three cultural bottles of the suspected lesions. RPMI-1640 media used in our studies have successful results either in isolation from suspected lesion or at the stage of diagnosis in patients. Amastigotes were in the first 1–5 days in the cultural media while promastigotes were seen up to 12 days; it was also observed that the highest level of production was seen in 9 and 10 days and it was determined that no growth existed in control bottles of the media (Figures 2 and 3). The results obtained from the two assays (microscopy and PCR) (Figures 4 and 5) for* Leishmania* were analyzed which showed some correlation between the two assays. The PCR sensitivity was 87.61% (99/113) of the total tested samples while the microscopy showed the 53.98% (61/113) sensitivity. Microscopy was positive in 61 samples for amastigotes of* Leishmania* presence as compared to PCR which detected kDNA of* L. tropica* in 99 samples, while culture showed 46.90% (53/113); thus the sensitivity of PCR is very high as compared to microscopy and culture. Microscopy and culture have poor sensitivity for low amastigotes in the field while PCR can amplify a single amastigotes if present and PCR was more specific than microscopy in terms of diagnosis (Table 3).

Molecular characterization of* Leishmania* species which cause cutaneous leishmaniasis in Kohat, Khyber Pakhtunkhwa, was reported in the present study. Sporadicalness of CL is reported in other parts of Pakistan, Afghanistan, India, and Iran where both* L. tropica* and* L. donovani* were found to cause leishmaniasis in particular areas [8, 19]. CL is prevalent in Pakistan and reported from all the provinces [16, 20] where the causative agent is* Leishmania tropica* [17, 21].

Our study revealed that* L. tropica was the sole causative agent for* cutaneous leishmaniasis which was examined in all cases in the endemic region in Kohat of Khyber Pakhtunkhwa. Out of 113 cases studied most belonged to a poor socioeconomic class, living in rural and hilly tract areas. The infection was more common in female than in male, which is in contrast to the report of [22, 23] and this may be due to ecological, cultural, environmental, and socioeconomic conditions of the areas. The finding of the present study was similar to the report of [8, 16].

This study emphasized diagnosis and species identification in a large number of patients with CL in the regions of Kohat, and it was identified the* L. tropica is the etiological agent of the infection*. The kDNA PCR was the most accurate diagnostic assay and was recognized as the most reliable method in the diagnosis of CL. The test may be standardized for the detection of parasite in patients of CL having negative microscopic examination and/or culture results. For the vitro culture and direct microscopic diagnosis a high number of amastigotes of CL are required which is very low in skin lesions [20, 23].

As in the specified region there is no facility for culture only diagnosis and detection of* Leishmaniasis* are based on direct microscopy which is easy and cheap with low accuracy even if carried out by skilled persons [24, 25]. While PCR is more sensitive, and specific than conventional methods in term of the diagnosis of the cutaneous leishmaniasis [8, 20]. So in the present study, culture showed 46.90% (53/113) sensitivity and direct microscopy with smear biopsies showed 68.14% (61/113) sensitivity, whereas PCR 87.61% (99/113) sensitivity which is more sensitive in diagnosis of the disease. Similarly tissue smears (37%–60%) and culture 38 percent results have been shown in CL in India and in the nearer countries of Pakistan [19, 26].

## Figures and Tables

**Table 1:** Comparative detection of *Leishmania* in culture, direct microscopy, and PCR *n* = 113.

Sample	Culture	Microscopy	PCR
Positive	53	61	99
Negative	60	52	14
Positive control	+	+	+
Negative control	−	−	−

**Table 2:** Days detected for observation of promastigotes in the culture media of RPMI-1640 from suspected lesions.

Media	1st day	2nd day	3rd day	4th day	5th day	6th day	7th day	8th day	9th day	10th day	11th day	12th day
RPMI 1640	−	−	−	+	+	+	+	++	++	++	+	+
Negative control	−	−	−	−	−	−	−	−	−	−	−	−

(−) indicates amastigote.

(+) indicates promastigotes.

(++) indicates promastigotes at peak level.

**Table 3:** Result showing the sensitivity and specificity of PCR, microscopy, and culture diagnostic methods.

Serial number	Methods	Sensitivity	Specificity
1	PCR	87.61%	12.38%
2	Microscopy	53.98%	46.0%
3	Culture	46.90%	53.09%
